# Modulation of the Leptin Receptor Mediates Tumor Growth and Migration of Pancreatic Cancer Cells

**DOI:** 10.1371/journal.pone.0126686

**Published:** 2015-04-28

**Authors:** Alisha M. Mendonsa, Madeleine C. Chalfant, Lee D. Gorden, Michael N. VanSaun

**Affiliations:** 1 Department of Cancer Biology, Vanderbilt University Medical Center, Nashville, Tennessee, United States of America; 2 Department of Surgery, Vanderbilt University Medical Center, Nashville, Tennessee, United States of America; 3 Department of Surgery, Division of Surgical Oncology, University of Miami, Sylvester Cancer Center, Miami, Florida, United States of America; University of Florida, UNITED STATES

## Abstract

Obesity has been implicated as a significant risk factor for development of pancreatic cancer. In the setting of obesity, a systemic chronic inflammatory response is characterized by alterations in the production and secretion of a wide variety of growth factors. Leptin is a hormone whose level increases drastically in the serum of obese patients. High fat diet induced obesity in mice leads to an overall increased body weight, pancreatic weight, serum leptin, and pancreatic tissue leptin levels. Here we report the contribution of obesity and leptin to pancreatic cancer growth utilizing an *in vivo* orthotopic murine pancreatic cancer model, which resulted in increased tumor proliferation with concomitant increased tumor burden in the diet induced obese mice compared to lean mice. Human and murine pancreatic cancer cell lines were found to express the short as well as the long form of the leptin receptor and functionally responded to leptin induced activation through an increased phosphorylation of AKT473. In vitro, leptin stimulation increased cellular migration which was blocked by addition of a PI3K inhibitor. In vivo, depletion of the leptin receptor through shRNA knockdown partially abrogated increased orthotopic tumor growth in obese mice. These findings suggest that leptin contributes to pancreatic tumor growth through activation of the PI3K/AKT pathway, which promotes pancreatic tumor cell migration.

## Introduction

Obesity and diabetes have been shown to be independent risk factors for the development of a number of epithelial cancers including pancreatic adenocarcinoma. Worldwide, obesity rates have risen at an unprecedented rate in the past decade[1]. Obesity complicated by the metabolic syndrome and type 2 diabetes mellitus are commonly comorbid conditions[2]. It has been suggested that diabetes may be linked to the development and progression of pancreatic cancer as 80% of pancreatic cancer patients experience some form of diabetes or altered insulin sensitivity[3]. Body mass index (BMI) and a 10 cm increase in waist circumference provided an increased relative risk of 1.11 for incidence of pancreatic cancer[4]. Additionally, murine models have demonstrated the importance of diet on the development of pancreatic cancers [5, 6].

Chronic obesity leads to alteration in the production and secretion of the adipokines, the cytokines secreted by the adipose tissue[7–12]. Leptin is one such adipokine which is dramatically increased in the obese patients[8, 9]. Leptin, typically known for its ability to regulate energy expenditure and satiety[13], binds to multiple isoforms of the leptin (ob; obese) receptor. The short form of the receptor is known to signal through PI3K/AKT pathway while the long form of the leptin receptor is known to signal through the JAK-STAT pathway and induce phosphorylation of STAT3[14–16]. Increased leptin has been reported in subsets of cancer patients and demonstrated to stimulate proliferation of colon cancer cells, breast cancer cell migration, glioma migration and invasion, as well as the growth of cholangiocarcinoma cells *in vitro*. [17–21]

The role of leptin and leptin receptor signaling in pancreatic cancer development and progression remains ill defined. Early studies demonstrated that *in vitro* treatment of pancreatic cancer cells with low levels of leptin induced a decrease in metabolic activity[22]. *In vitro* studies demonstrated growth and metastasis of a murine pancreatic cancer cell line was shown to be increased in genetically obese mice caused by loss of leptin or loss of the long isoform of the leptin receptor[23]. Tumoral adipocytes were also shown to be positively correlated with proliferation of pancreatic cancer xenografts implanted in obese mice[24]. Additionally, non-alcoholic fatty pancreatic disease (NAFPD) and steatopancreatitis were found represent a potentially significant risk factor for human pancreatic cancer[25].

To understand whether tumoral expression of leptin receptors regulated the growth of pancreatic cancers in the setting of obesity, we orthotopically injected pancreatic cancer cells in lean and obese mice utilizing interference RNA technology to deplete the leptin receptor from pancreatic cancer cells. Results demonstrate that leptin receptor expression potentiates pancreatic tumor growth independent of tumor cell proliferation.

## Materials and Methods

### Ethics Statement

This study was performed in accordance with the Guide for the Care and Use of Laboratory Animals of the National Institutes of Health and the approval of the Vanderbilt Institutional Animal Care and Use Committee/Office of Animal Welfare Assurance (M/12/277). Animal housing and care was in accordance with an accredited laboratory animal facility. All procedures were performed in accordance with approved methods to reduce the number of animals and any potential animal discomfort.

### Mice and Diet Manipulation

Eight week old C57bl/6J male mice were obtained from Jackson Research Laboratories, separated into appropriate cages and fed either a lean diet of 13.5% fat (5001, LabDiet: 13.5% calories from fat, 58% from carbohydrates, and 28.5% from protein) or fed an obesity inducing diet of 42% fat “DIO, diet induced obesity” (TD.88137, Harlan Teklad: 42% calories from fat, 42.7% from carbohydrates, and 15.2% from protein) ad libitum. Body weight and total pancreatic weight was measured after three months on diet. Plasma was collected from mice and leptin levels were assessed through a mouse specific Quantikine assay according to the manufacturer’s protocol (R&D Systems).

### Orthotopic tumor growth

Pancreatic cancer cells were harvested via Trypsin/EDTA when 80% confluent. Cells were resuspended into pharmaceutical grade saline and injected orthotopically into the tail of the pancreas of lean and diet induced obese mice to establish tumors. In brief, mice were anesthetized with isoflurane via inhalation and a paramedian incision was made in the abdomen. The tail of the pancreas was externalized and 2.5x10^5^ pancreatic cancer cells were resuspended in 25μl of veterinary saline and injected into the tail of the pancreas using a 27 gauge needle. An immediate bubble ensured an accurate injection and a cotton swab applied during withdrawal of the needle ensured that cells were not spilled into the abdomen. The pancreas was then carefully placed back into anatomical position and the mice were sutured and allowed to recover under IACUC approved monitoring. Tumors were allowed to grow to an endpoint of 28 days and mice were euthanatized via carbon dioxide overdose. Tumors were excised along with the remaining pancreas, weighed, fixed in 4% paraformaldehyde overnight, and then processed for histological analysis. Hematoxylin/eosin or trichrome stained sections were then scanned at 20x magnification with an Ariol SL-50 scanner (Leica). Tumor area, adipocyte number and adipocyte size was calculated at tumor midpoint using Ariol images in association with Digital Image Hub analysis software (Leica). For bioluminescent imaging analysis, in vivo imaging was performed the day after injection and each subsequent week via an IVIS 200 system (Xenogen). Bioluminescence was measured for each mouse on each day using the same exposure settings. Quantitation of total flux was calculated with Living Image analysis software by creating a predefined region of interest which was kept constant for all mice. Immunohistochemical analysis for Ki67 (1:250, Abcam Ab15580) and appropriate secondary/tertiary was used to determine the number of proliferating cancer cells. Ki67 staining was quantified by assessing the total number or the percent area of positively stained nuclei within the tumor. Post-acquisition analysis and statistical analysis was performed using GraphPad Prism analysis software.

### Cell Lines

Standard previously published cell culture methods were used to maintain pancreatic cancer cell lines, including murine Panc02 (NIH repository, DTP/DCTC/NCI[26]), PanIN 4313 [27], K8484 and DT8082[28] as well as human MiaPaca-2, Panc1, and BXPC-3 (ATCC; CRL-1420, CRL-1469, CRL-1687). Luciferase tagged cell lines were generated by infecting the cell lines with a commercial lentiviral titer for firefly-luciferase (Genecopoeia, LPP-FLUC-LV105-025 containing 1x10^8^ TU/ml). Non-infected cells were eliminated after addition of puromycin (10ug/ml, Sigma). Luciferase expression was confirmed for each line through One-Glo analysis (Pierce).

### Realtime PCR Analysis

RNA was extracted from each cell line using a combined Qiazol extraction and Qiagen RNeasy mini kit purification. One microgram of total RNA was reverse transcribed using the high capacity cDNA reverse transcription kit (Applied Biosystems). Real-time PCR was performed using specific primers for the murine leptin receptor; QT00154133, QT01045380, and QT01045373 (Qiagen) in combination with the iQ SYBR green supermix kit (Bio-Rad) according to the manufacturer's instructions in a CFX96 real time PCR detection system (Bio-Rad). Each transcript in each sample was assayed three times and the fold-change ratios between experimental and control samples for each gene used in the analysis were calculated using 18S levels as a reference. Human leptin receptor quantitative primers were as follows for a common region of the leptin receptor (forward: 5’TTGTGCCAGTAATTATTTCCTCTT, reverse: 5’CACACCAAAGAATGAAAAAGCTAT), the short form of the leptin receptor (forward: 5’TTCCTGGGCACAAGGACTTA, reverse: 5’GCTCCAAAAGAAGAGGACCA), and the long form of the leptin receptor (forward: 5’TTCCTGGGCACAAGGACTTA, reverse: 5’TTTGTGTCCCTGGGTACTTGA). Human leptin receptor primers for RT-PCR used a common forward primer 5’CGTGCAGTCACTCAGTGCTTA and reverse primers to detect the short form 5’TTTGTGTCCCTGGGTACTTGA or the long form 5’GCTCCAAAAGAAGAGGACCA. Control primers for human beta actin were forward 5’GAGCACAGAGCCTCGCCTTT and reverse 5’ATCCTTCTGACCCATGCCCA.

### Edu proliferation assay

Proliferation was assessed through the use of an EdU flow cytometric-based analysis. Briefly, 1x10^5^ cells were plated in each well of a 24 well plate and allowed to attach overnight. Cells were then serum starved for 8h, followed by appropriate treatments for an additional 24h. EdU was added to the culture medium at 25μM during the last 3 hours of treatment for incorporation. At the end of treatment, cells were released with trypsin/EDTA and then washed with PEB (PBS/2mM EDTA/1% BSA). Cells were fixed with 2% buffered formalin for 30 minutes at room temperature, spun at 300xg, aspirated and then rinsed in PBS containing 1% BSA. Cells were permeabilized with addition of 0.25% triton for 15 minutes, pelleted at 450xg, and then washed with PBS/BSA and pelleted again. Cells were labeled in a reaction mixture containing 150mM Tris PH 8.5, 1.5mM CuSO4, 10mM 647-azide, and 100mM ascorbic acid (added in order) for 15 min at room temperature in the dark. The reaction was stopped by 5 fold addition of PEB buffer. Cells were labeled with 1ug/mL propidium iodide and pelleted at 450xg. Cells were resuspended in 250μl of PEB and then quantified by flow cytometry on an Accuri C6 cytometer. Cells were gated by SSC vs FSC and measured for the percentage of PI positive cells within the EdU-647 positive value gate.

### Migration assay

Cell migration was assessed by a scratch assay. Pancreatic cancer cells (2.5×10^5^ cells/well) were seeded in complete media and allowed to adhere overnight and reach confluence. Upon confluence cells were serum starved for 8 hours. Cells were then scratched with a pipet tip to produce a wound free of cells and media was changed to the appropriate treatment conditions. Leptin was administered at 250ng/mL with and without the addition of LY294002 (20μM, Sigma) and cultured for an additional 24 h at 37°C in 5% CO_2_. The scratch width was recorded immediately after the scratch and again at 24 hours after the scratch. The scratch width was calculated at three positions in each well and expressed as an average and then measured in four experiments.

### Western Analysis

Lysates from pancreatic cancer cell lines were collected in ice cold RIPA buffer (50mM Tris pH 7.4, 50mM HEPES, 150mM NaCl, 5mM EDTA, 0.2% SDS, 10mM NaF, 1mM Na_3_VO_4_, 0.5% Sodium Deoxycholate, and 1% Triton X-100) with broad spectrum cOmplete Mini protease inhibitor cocktail (Roche) as well as phosphatase inhibitor cocktail (Cell Signaling, 5870S). Lysates were briefly sonicated, spun at 10000xg for 15min and total protein concentration in the supernatant was measured via BCA protein analysis (Pierce). Control lysate COLO 320DM was obtained from Santa Cruz (sc-2226). 25μgs of lysates were separated via SDS-PAGE, transferred to nitrocellulose, blocked with 3% BSA and then blotted with primary antibody: LepR (SantaCruz (K-20), sc-1835 1:250 and (H-300) sc-8325 1:250), pAKT473 (Cell Signaling, 4060S 1:1000), tAKT (Cell Signaling, 4821S 1:1000), pSTAT3 (Cell Signaling, 9145L 1:1000), tSTAT3 (abcam, ab5073 1:1000), pAMPK (Abcam, Ab133448 1:1000), tAMPK (Cell Signaling, 2603S 1:1000), and Actin (Abgent, AM1829b 1:3000). Appropriate secondary antibodies conjugated to IR680 and IR800 (Rockland 1:10000) were used in conjunction with Odyssey Image acquisition and densitometric analysis (Li-Cor Biosciences). Densitometry for pAKT was compared to tAKT levels and pSTAT3 was compared to tSTAT3 levels, which were then respectively compared to the zero timepoint or the parental control. Densitometry for control and leptin receptor knockdown lines were compared to actin and then normalized to the parental levels.

### shRNAmir Knockdown

Lentiviral titers were produced from GIPZ vector constructs from multiple regions of murine leptin receptor (V2LMM 70436 and V2LMM 190793, ThermoScientific) or for a control vector (RHS4480) using the Trans-Lentiviral packaging system according to the manufacturer’s protocol (Open Biosystems). Lentiviral titer supernatants were concentrated using Lenti-Pac concentration solution and 2.5x10^5^ pancreatic cancer cells were transduced according to manufacturer’s recommended protocol (Genecopoeia). After transduction, positive cells were isolated under 10μg/ml puromycin selection and confirmed visually for GFP expression. Additionally, knockdown was confirmed for mRNA via qPCR and for protein via western blot analysis.

## Results

### Diet induced obesity contributes to pancreatic adiposity

**Fig 1:**
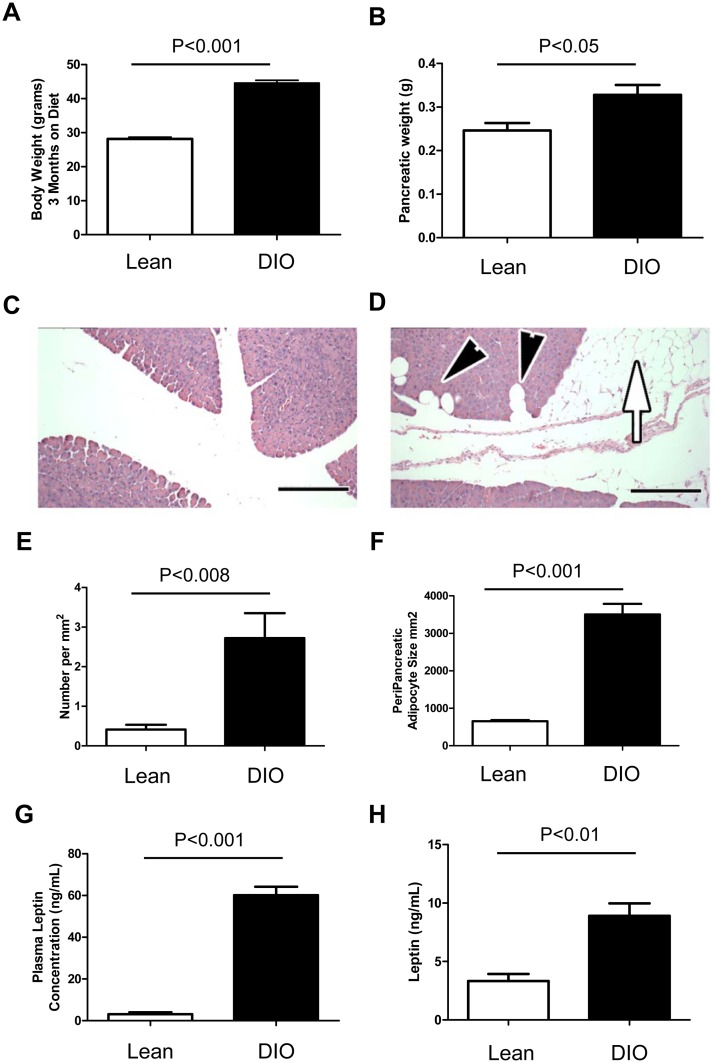
High fat diet induced obesity contributes to pancreatic adiposity in mice. Mice were maintained on a 42% high fat diet for 3 months to induce obesity. DIO mice gained significantly more total body weight (A) as well as pancreatic weight (B). Histological analysis of total pancreata from Lean (C) compared to DIO mice (D) showed accumulation of interpancreatic (white arrow) and intrapancreatic (black arrowheads) adipose in DIO pancreas. The total number of intrapancreatic adipocytes (E) as well as the size of peripancreatic adipocytes (F) was greater in the DIO pancreas. Leptin levels were increased in both plasma samples and pancreatic tissue of obese mice (G). Scale bars are 100μm.

It has previously been reported that consumption of a high fat diet leads to obesity, insulin resistance and increased leptin levels by eighteen weeks of age in mice[29]. Additionally, it has been shown that the human pancreas itself is subject to fatty infiltration, identified as fatty pancreatic disease[25]. To determine whether diet induced obesity could result in fatty infiltration of the murine pancreas, mice were maintained on a high fat diet for three months. The animals showed a dramatic increase in total body weight as well as pancreatic weight in the diet induced obese (DIO) mice (Fig 1A and 1B). Average fixed and dry pancreatic weight for lean mice was 0.221g compared to 0.325g for obese mice. Histological examination of pancreata from lean and diet induced obese mice showed the presence of both interpancreatic and intrapancreatic adipose in the DIO mice (Fig 1C and 1D). The number of adipocytes per mm^2^ of acinar tissue as well as the size of the peripancreatic adipocytes was significantly increased in the DIO pancreas (Fig 1E and 1F). Additionally, we determined the mice had a 20 fold increase (average for lean was 3.155ng/mL and DIO was 60.156ng/mL) in plasma leptin levels after twelve weeks on diet (Fig 1G). Normal leptin levels are typically found at 1–10ng/ml in human serum and 10–50ng/ml in obese patients[30]. In the setting of increased pancreatic adipose, the relative tissue levels of leptin in the pancreas were found to be increased in DIO pancreata compared to lean pancreata (Fig 1H).

### Diet induced obesity increases orthotopic pancreatic tumor growth

**Fig 2:**
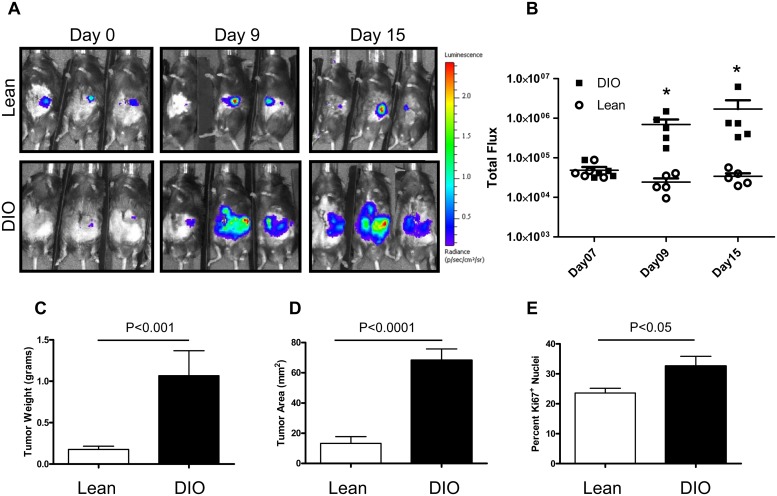
Diet induced obesity increases orthotopic Panc02 pancreatic tumor growth. Bioluminescence revealed increased growth over time in DIO mice compared to lean mice (A). Total flux from luciferase imaging was statistically different by Day09 after orthotopic tumor cell injection (B). Endpoint tumor weight (C) as well as tumor area (D) were significantly increased in the DIO mice. Proliferation assessed by Ki67 staining was increased in DIO tumors compared to lean tumors (E). Radiance heat map scale is x10^5^ p/sec/cm^2^/sr. Statistical analysis by Mann-Whitney of p<0.0079 (*).

Obesity is a significant risk factor for many cancers and has been shown to potentiate their growth in mice [5, 31, 32]. To examine whether diet induced obesity was sufficient to increase the growth of pancreatic cancer *in vivo*, we utilized a murine syngeneic orthotopic pancreatic cancer model. We have previously shown that diet induced obesity increased the number of hepatic metastases in a splenic injection model of colon cancer metastasis in the setting of fatty liver disease[33]. Using an EL-Kras model it has been shown that obesity prone C57BL/6J mice fed omega-6 fat have an earlier onset and an increased frequency of PanIN, pancreatic intraepithelial neoplasia, lesions[5]. Therefore, we postulated that diet induced obesity and the development of NAFPD in mice may also increase the growth of pancreatic cancers. C57BL/6J mice were placed on a high fat diet for three months to induce obesity. Panc02 pancreatic adenocarcinoma cells were then injected orthotopically into the tail of the pancreas and monitored for growth. In vivo primary tumor growth was monitored using bioluminescence (IVIS) imaging analysis in combination with luciferase labeled Panc02 cells (Fig 2A). Panc02 tumors orthotopically implanted in the diet induced obese mice showed a significant increase in total photon flux after nine days of growth and a continued growth over time while tumors in the lean mice showed slow growth over the same time period (Fig 2B). Ex vivo analysis at 28 days post injection confirmed larger tumor growth in the diet induced obese mice compared to the lean mice calculated as total tumor weight (Fig 2C). Ariol scanning with computational analysis also showed an increased tumor area in obese mice (Fig 2D). Endpoint tumors were stained for the proliferation marker Ki67 and showed significantly increased tumor cell proliferation in DIO mice compared to lean mice (Fig 2E).

### Pancreatic cancer cell lines express functional leptin receptors

**Fig 3:**
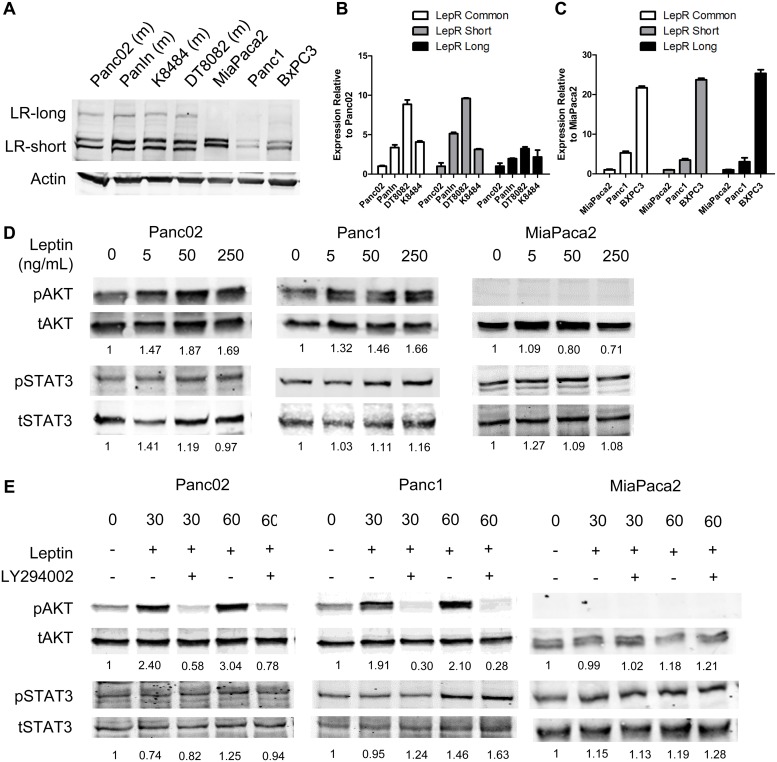
Pancreatic cancer cell lines express functional leptin receptors. Western and real-time qPCR analysis verified leptin receptor expression for the long and short forms of the leptin receptor in murine and human pancreatic cell lines (A,B,C). Western anlayis demonstrated stimulation of pancreatic cancer cell lines with leptin lead to the phosphorylation of AKT in the Panc02 and Panc1 cell lines, and to the phosphorylation of STAT3 (D). Addition of the PI3K/AKT inhibitor LY294002 was able to block leptin induced phosphorylation of pAKT but did not affect leptin induced pSTAT3 activation in the Panc1 cell line. Densitometric analysis was used to quantify the amount of pSTAT3 and pAKT relative to total levels for each protein and then normalized to zero timepoint.

Studies have demonstrated that pancreatic β-cells express functional leptin receptors[34], yet the receptor expression levels and function in pancreatic cancer has not been addressed. To determine whether pancreatic cancer cells expressed leptin receptors, we isolated RNA and protein from multiple human and murine pancreatic cancer cell lines. Western blot analysis was performed to determine the relative protein level of leptin receptors in our panel of pancreatic cancer cell lines. Both the short isoform (LR-short) and the long form (LR-Long) were present in pancreatic cancer cell lines, yet the long form in human lines was only weakly detected using the K-20 antibody (Fig 3A) Presence of the long leptin receptor isoform was additionally verified using the H-300 antibody (S1B Fig). Both forms were additionally detected through PCR analysis in murine as well as human cell lines (Fig 3B and 3C and S1A Fig). Cell lines were treated with exogenous leptin at 5ng/mL, 50ng/mL, and 250ng/mL to determine the extent of pAKT and pSTAT3 activation. Western analysis coupled with densitometry, showed that leptin induced activation of pAKT-S473 in Panc02 and Panc1 lines, but not in the MiaPaca cell line (Fig 3D). Leptin induced activation of pAKT was further suppressed by the PI3K inhibitor LY294002 at 30 and 60 minutes (Fig 3E). Leptin stimulated pSTAT3 in the human Panc1 cell line with increasing concentration, although lower concentrations were more effective in inducing pSTAT3 in the the murine Panc02 line and in the MiaPaca2 cell lines (Fig 3D). These results demonstrate that pancreatic cancer cells express functional leptin receptor, yet ligand stimulation of either pAKT or pSTAT3 is dependent on the type of pancreatic cancer cell line.

### Leptin stimulates proliferation of murine pancreatic cancer cells

**Fig 4:**
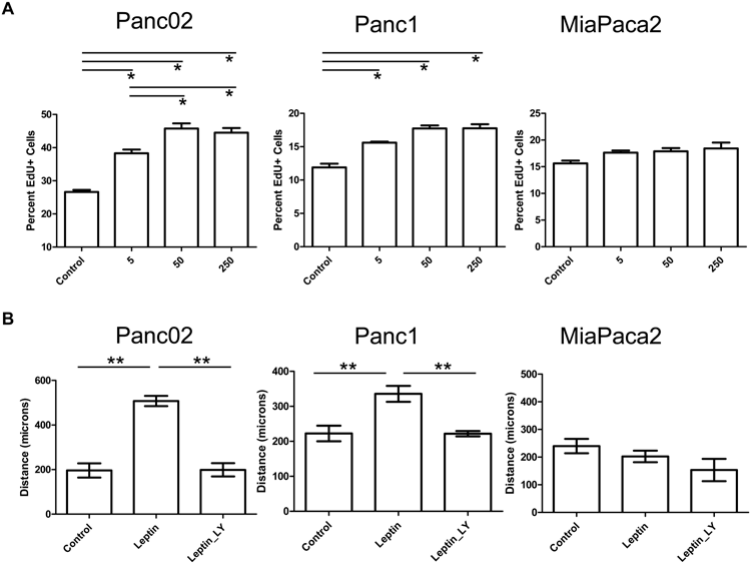
Leptin induced proliferation and migration of pancreatic cancer cells is cell line dependent. Leptin stimulation at 5, 50, and 250ng/ml caused an increase in proliferation assessed through EdU incorporation in murine Panc02 and human Panc1 cell lines but did not alter proliferation in human MiaPaca cells (A). Leptin stimulation caused an increase in migration recorded as distance migrated for Panc02 and Panc1 cells assessed through scratch assay which was blocked by PI3K inhibitor LY294002 (B). Statistical analysis by *ANOVA of p<0.0001, and **T-test of p<0.05.

Leptin has been shown to stimulate proliferation in a variety of cancer cell lines[21, 35–38]. The increased level of leptin in the plasma as well as the pancreas tissue suggested that leptin might be involved in pancreatic tumor growth. Activation of pAKT or pStat3 has been associated with leptin induced proliferation, survival, immune tolerance and invasion in cancer cells[35, 36, 39–42]. Contrary to multiple studies showing activation of proliferation in cancer cells upon leptin stimulation, the treatment of MiaPaca or Panc1 cells with leptin was reported to cause a decrease in their metabolic activity via an MTT assay[22]. Due to the increased pAKT and pSTAT3 observed in our studies with leptin treatments, we were interested to determine how leptin stimulation altered the proliferation of pancreatic cancer cell lines. Edu incorporation assays were performed in order to determine whether leptin effected the proliferation of pancreatic cancer cells (Fig 4A). Leptin stimulation showed a significant increase in proliferation via Edu incorporation for the murine Panc02 cell line and the human Panc1 cell line at all concentrations tested, yet it did not alter proliferation in the MiaPaca cell line at any concentration tested.

### Leptin increases migration of pancreatic cancer cells

Aside from proliferation, leptin has also been shown to increase the migratory capacity of cancer cells[18, 19, 43, 44]. Histological analysis of the tumors in obese mice showed a high degree of tumor cell infiltration into the peri-pancreatic adipose in the DIO mice compared to the lean mice (data not shown). This suggested that the pancreatic cancer cells might be responding with enhanced motility and migration to cytokines or adipokines released by the adipose tissue. To determine whether leptin might additionally act as a migratory factor for pancreatic cancer cells we performed scratch assays. Scratch assays demonstrated that leptin significantly increased the migration of both Panc02 as well as Panc1 cells in vitro (Fig 4B), while MiaPaca cells did not show migratory activation in response to leptin. Addition of the PI3K/AKT inhibitor LY294002 blocked leptin induced migration of pancreatic cancer cells.

### Knockdown of leptin receptor

**Fig 5:**
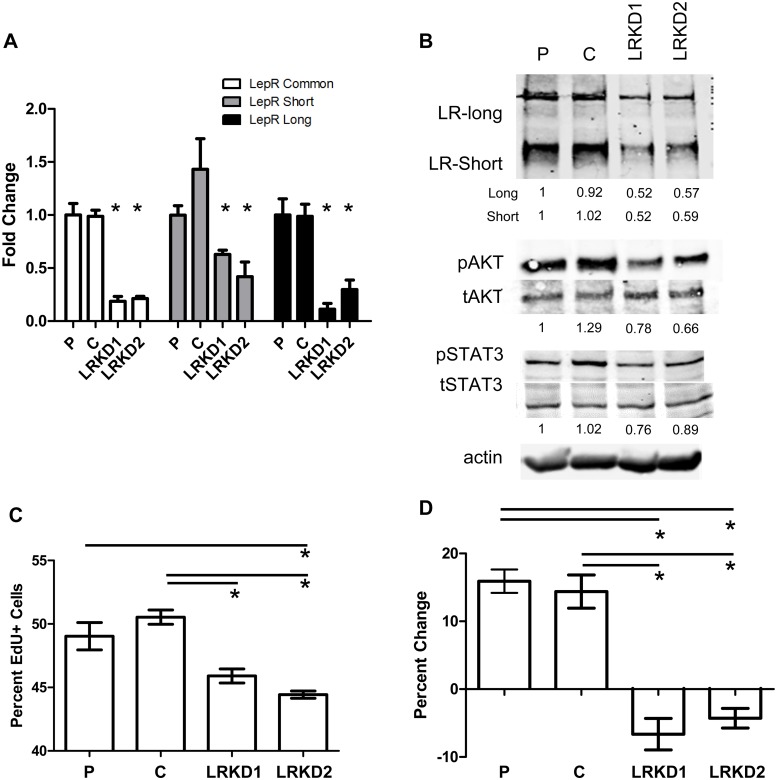
Knockdown of leptin receptor in pancreatic cancer cells leads to functional impairment of leptin signaling. Long and short leptin receptor RNA levels measured through realtime PCR analysis were both significantly decreased using two different shRNAmir lentiviral titers in the Panc02 cell line (A). Western and densitometric analysis confirmed leptin receptor knockdown as well as reduced activation of pAKT (B). Basal proliferation was significantly decreased in both the LRKD1 and LRKD2 knockdown cell lines when grown in serum free conditions (C). Stimulation with leptin at 50ng/mL induced proliferation in parental and control cells but failed to induce proliferation (percent change compared to untreated) in either of the knockdown cell lines compared to parental or control shRNA (D). Statistical analysis by ANOVA *represents p<0.014 short, p<0.0023 long, p<0.0003 common (A); ANOVA p<0.0008 (C).; ANOVA p<0.0001 (D). *p<0.05 in C,D.

In order to determine the contribution of the leptin receptor to pancreatic cancer growth in obese mice, we knocked down the expression of the leptin receptor using lentiviral shRNAmir based techniques in the murine Panc02 cell line. We were able to obtain a significant reduction in the RNA expression of both the long as well as the short forms of the leptin receptor using two different shRNAmir constructs, LRKD1 and LRKD2 (Fig 5A). Protein analysis via western blot and densitometric analysis confirmed the knockdown effect with both constructs (Fig 5B). Additionally, knockdown of the leptin receptor also conferred a decrease in the activation levels of pAKT and pSTAT3 (Fig 5B). Using an Edu based proliferation assay we were able to show that the knockdown lines had a lower basal proliferative rate in serum free medium (Fig 5C). Stimulation of both parental and control shRNA Panc02 cells with leptin induced an increase in proliferation that did not occur in the LRKD1 or LRKD2 Panc02 cells (Fig 5D).

### Knockdown of the Leptin receptor abrogates DIO associated tumor growth

**Fig 6:**
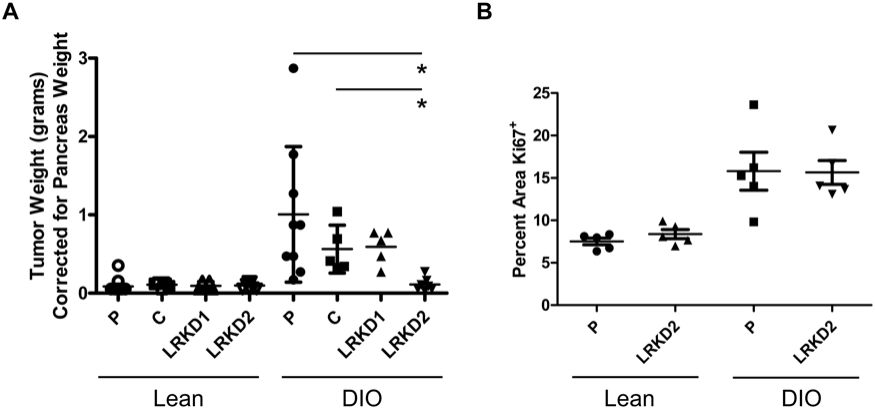
Leptin receptor knockdown in Panc02 cells antagonizes obesity stimulated orthotopic tumor growth in mice. Panc02 cells with leptin receptor knockdown variants LRKD1 and LRKD2 were orthotopically injected into lean and DIO mice. (A) Differences between parental (P), control shRNA (C), and knockdown variants were not statistically different when comparisons were made between control and knockdown tumors in lean mice. Leptin receptor knockdown variant LRKD2 showed a significantly decreased tumor weight in DIO mice when compared to the parental and control tumors in DIO mice. (ANOVA p<0.0001, *p<0.05). (B) Assessment of tumor cell proliferation through Ki67 staining was not different between control DIO tumors compared to LRKD2 DIO tumors, but was significant between lean and DIO tumors (ANOVA p<0.0004, *p<0.05).

Leptin receptor knockdown Panc02 cells were used to determine whether the in vivo pancreatic orthotopic tumor growth increase observed in obese mice was due to increased leptin signaling in DIO mice. Leptin receptor knockdown Panc02 cell lines were injected orthotopically into lean and DIO pancreata. After twenty-eight days of growth, mice were sacrificed and tumors were collected and weighed. Both of the leptin receptor knockdown lines showed reduced tumor weights in the DIO mice, yet only LRKD2 showed a statistically decreased tumor weight when compared to the parental or the control shRNA Panc02 cell lines grown in the DIO mice (Fig 6A). Knockdown tumors in lean mice were not significantly different from each other. Proliferation of tumors from LRKD2 were compared to parental tumors to determine the number of Ki67 positive cells, which revealed similar levels of proliferation in the wildtype DIO tumors compared to the leptin receptor knockdown DIO tumors (Fig 6B). Therefore, the difference in tumor growth did not correlate with tumor cell proliferation.

## Discussion

Obesity and alterations in diet composition have been reported to affect the growth and onset of pancreatic cancers in multiple murine models[5, 6]. This study demonstrates that diet induced obesity correlates with the development of a fatty pancreas and that obesity potentiates the growth of pancreatic cancer. Diet induced obesity correlated with in an increased proliferation of orthotopically implanted pancreatic tumor cells *in vivo*. Additionally, fatty pancreas disease was shown to be associated with increased incidence of lymph node metastases [45]. Importantly, some of the noted risk factors associated with pancreatic cancer are obesity, chronic pancreatitis, and diabetes[46]. Our results are consistent with the general consensus that obesity is a contributing factor to increased pancreatic cancer growth and offer a contributing mechanism for enhanced tumor growth mediated by leptin induced changes in STAT3 and/or PI3K-AKT signaling.

Obesity leads to the development of both a local and systemic chronic inflammatory response[11, 12]. This chronic inflammatory response leads to an alteration in the production and secretion of adipokines as well as other cytokines and growth factors. Leptin has been widely publicized to be significantly increased in the serum of obese patients[8, 9]. We were able to identify that leptin is increased in the serum (approximately 5ng/mL in lean and 50ng/mL in obese) as well as in the pancreatic tissue of obese mice in our diet induced model. The local increase of leptin in the pancreas and the ability of leptin to induce proliferation of cancer cells, warranted a study to determine the contribution of leptin to pancreatic tumor growth in this murine model.

Leptin signals through the short as well as the long form of the leptin receptor. We were able to determine that murine and human pancreatic cancer cells express both forms of leptin receptors through mRNA and protein based analyses. Albeit, the mRNA expression and total protein levels for the different leptin receptor isoforms did not seem to be directly correlative. The short form of the receptor (LepRa) contains only the Jak2 binding site while the long form (LepRb) encodes the full length transcript containing additional tyrosine phosphorylation sites for interactions with STAT3, SHP2 and SOCS[47–50]. The long form and short forms of the leptin receptor are capable of activating IRS1 subsequent to Jak activation by leptin stimulation; further leading to PI3K, AKT and NF-kB pathway activation[51, 52]. Application of recombinant leptin to pancreatic cancer cells in vitro resulted in a significant increase in the phosphorylation of AKT,in both the murine Panc02 as well as the human Panc1 suggesting that the short form of the receptor was functional in these cells. Yet, human MiaPaca2 cells did not activate pAKT in response to leptin. Additionally, leptin stimulation led to an increase in the phosphorylation of STAT3 only in the human Panc1 cell line, suggesting unique functional response of the long form of the leptin receptor in these cells. Our results suggest that pancreatic cancer cells vary in their responsiveness to leptin. Further studies are still needed to determine the exact functional significance for stimulation of the long versus the short form of the leptin receptor.

Leptin was only able to increase the proliferation of the murine Panc02 cell line in vitro, yet leptin induced migration of both the Panc02 and the Panc1 cell lines that was dependent on activation of AKT. Inclusion of the inhibitor LY294002 and subsequent inhibition of leptin induced cell migration, which implies that leptin activation of motility is dependent upon the PI3K pathway. Therefore, these results suggest that the short form of the leptin receptor is predominantly activated in pancreatic cancer cells and that its activation affects tumor cell motility.

The role of leptin on pancreatic cancer growth in vivo had previously been reported through experiments utilizing the LepDB (long form leptin receptor deficient) and LepOB (ligand deficient) mice that inherently develop obesity. The authors demonstrated a larger tumor volume and an increased cellular uptake of BrdU in tumors grown in these mice[23]. Potential questions remaining from these studies were related to the role of the leptin receptor in the cancer cells themselves, because xenografted cancer cells maintain functional leptin receptors and the LepDB mice still possess a functional short form of the leptin receptor[53]. To answer some of the questions in this study, we utilized application of leptin receptor specific shRNA in the Panc02 cells that led to knockdown of both the long and short forms of the leptin receptor. Orthotopic injection of the knockdown cells into mice resulted in a decreased tumor size in the obese mice, but failed to demonstrate a difference in tumor cell proliferation. We believe that the loss of leptin signaling to overall signaling of the STAT3 and/or PI3K pathway is likely augmented by increased IL-6, TNF-α or IGF in the obese state. Leptin can additionally affect the recruitment of inflammatory mediators as well as to affect the vascular system, aspects that were not addressed in this study. Use of leptin receptor deficient mice in the murine KrasG12D genetic model system may provide more information on these aspects of tumor development in the absence of leptin.

In conclusion, we demonstrate that leptin is partially responsible for the growth of pancreatic tumors in obese mice. Although leptin was capable of stimulating Panc02 and Panc1 cell proliferation in vitro, leptin does not appear to significantly contribute to the tumor cell proliferation in vivo. In vitro, leptin stimulation directly led to the phosphorylation of AKT, which was necessary to confer increased pancreatic cancer cell motility. Leptin induced motility of tumor cells may therefore be responsible for increased aggressiveness of select pancreatic cancers in the setting of obesity. It will be important to determine the role of leptin in a larger panel of human cell lines as well as patient derived xenografts in order to fully understand its effect on human tumor progression.

## Supporting Information

S1 Fig Leptin receptor expression in human cell lines. (A) The long as well as the short forms of the leptin receptor were detected using PCR based analysis. Beta actin was used as a positive control for mRNA. (B) The long form of the leptin receptor was detected using an anit-human leptin receptor antibody H-300. COLO320DM cell lysate was used as a positive control for the long form of the human leptin receptor.
(TIF)
