# Supplementary figures and images for: Modulation of the Leptin Receptor Mediates Tumor Growth and Migration of Pancreatic Cancer Cells
Source: PLoS One. 2015 Apr 28;10(4):e0126686. doi: 10.1371/journal.pone.0126686 (PMC4412670; doi:10.1371/journal.pone.0126686)

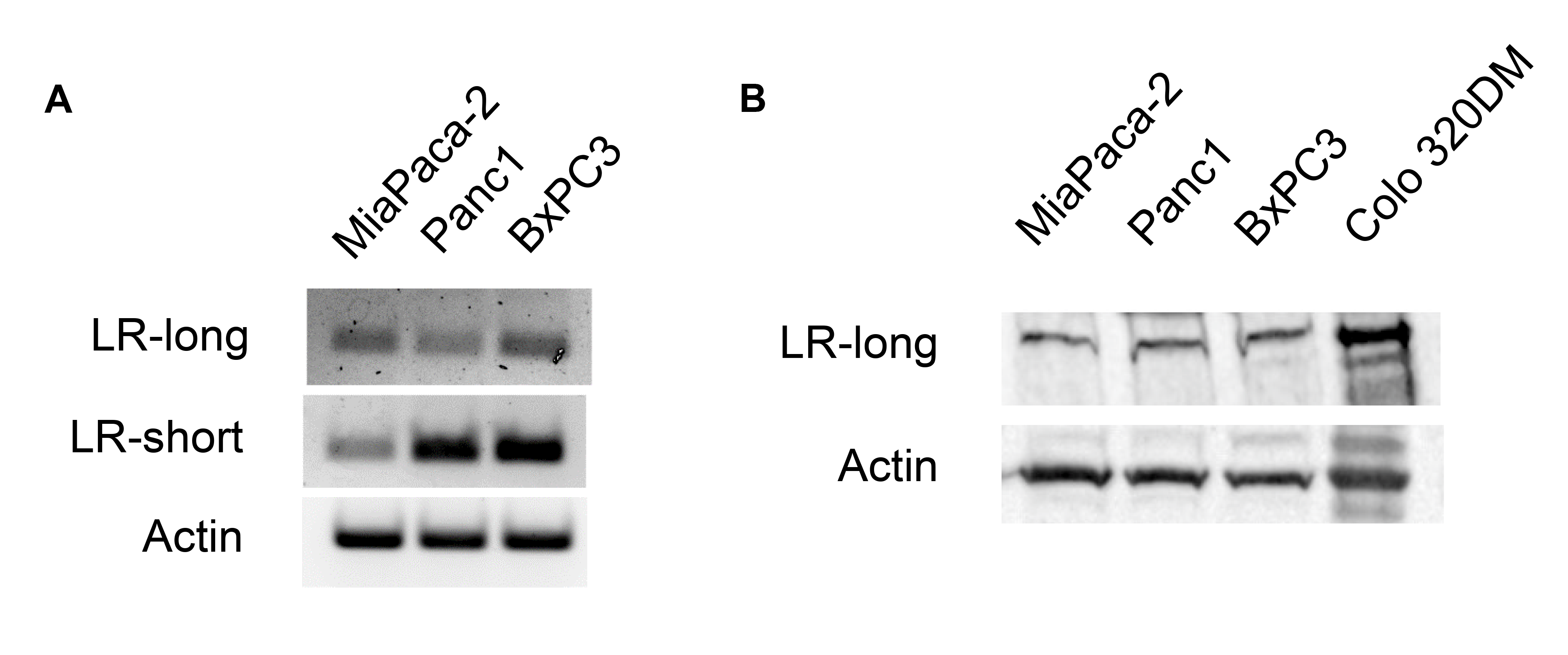

Supplement: S1 Fig — (A) The long as well as the short forms of the leptin receptor were detected using PCR based analysis. Beta actin was used as a positive control for mRNA. (B) The long form of the leptin receptor was detected using an anit-human leptin receptor antibody H-300. COLO320DM cell lysate was used as a positive control for the long form of the human leptin receptor. (TIF) [file pone.0126686.s001.tif]
